# Associations of Real-Time Ultrasound and Strain and Shear Wave Elastography with Gastrointestinal Organs: A Systematic Review

**DOI:** 10.3390/diagnostics13213302

**Published:** 2023-10-25

**Authors:** Nismat Javed, Haider Ghazanfar, Abhilasha Jyala, Harish Patel

**Affiliations:** 1 Department of Internal Medicine, BronxCare Health System, Bronx, NY 10457, USA; nismatjaved@gmail.com; 2 Department of Gastroenterology, BronxCare Health System, Bronx, NY 10457, USA; haidergh@gmail.com (H.G.); ajyala@bronxcare.org (A.J.)

**Keywords:** elastography, liver, pancreas, thyroid, prostate, lymph node, diagnosis, outcomes

## Abstract

Ultrasound elastography is gaining attention for its diagnostic potential across various medical fields, and its physical properties make it valuable in modern clinical medicine. However, its specific attributes, especially in the context of recent medical advancements, remain relatively unexplored. This study aimed to identify instrument-specific characteristics and applications of real-time ultrasound elastography, shear wave elastography, and strain elastography, particularly within gastroenterology. Following PRISMA guidelines, the study examined elastography articles on databases like PubMed, resulting in 78 included articles. Data on patient demographics, organ involvement, specificity, sensitivity, accuracy, positive predictive value, and negative predictive value were extracted. Statistical analysis involved SPSS version 21, with significance set at *p* < 0.05. The majority of patients were male (50.50%), with a mean age of 42.73 ± 4.41 years. Shear wave elastography was the most prevalent technique (48.7%), and liver investigations were predominant in gastroenterology (34.6%). Gastrointestinal applications showed higher sensitivity, positive predictive value, and negative predictive values (*p* < 0.05) but lower specificity (*p* < 0.05). Real-time ultrasound elastography exhibited increased specificity, accuracy, and predictive values (*p* < 0.05). Ultrasound elastography appears more accurate and effective in gastroenterological settings. Nonetheless, its performance depends on instrument-specific and operator-dependent factors. While promising, further studies are necessary to ascertain optimal utilization in both gastrointestinal and non-gastrointestinal conditions.

## 1. Introduction

Ultrasound elastography, a technique from the 1990s, has gained widespread attention in recent years [1]. Given its properties of elasticity, the modality has been modified to allow for quantification of many characteristics of diseases [2]. With the different methods of wave propagation (longitudinal and perpendicular), the elastic modulus varies, leading to multiple radiological variations that can be applied [2]. Ultrasound elastography is further divided into shear, strain, and acoustic force elastography and real-time tissue elastography. Shear wave elastography uses an ultrasound transducer to generate and propagate shear waves within the tissue being examined. These waves are essentially waves of deformation that travel through the tissue. The equipment measures the speed at which these shear waves travel through the tissue. The velocity of shear wave propagation is directly related to the tissue’s stiffness. Stiffer tissues transmit the shear waves faster, while softer or more elastic tissues transmit them more slowly. This information about differences in velocity is then utilized to create an elastogram [2]. Real-time elastography is often performed using an ultrasound transducer, that emits high-frequency sound waves into the body, and the resulting echoes are used to create images of the underlying tissues. The technique uses specialized software to analyze the deformation of the tissue in response to natural physiological motion (such as cardiac pulsations or respiratory movements). This deformation is linked to tissue elasticity, with stiffer tissues deforming less than softer ones. This information is then displayed simultaneously with the ultrasound images.

Strain elastography is a technique used to evaluate the elasticity or rigidity of bodily tissues. Its primary objective is to offer additional insights into tissue characteristics for the purpose of diagnosing and monitoring various medical conditions. In this method, a specialized probe or transducer is employed to apply force or stress to the target tissue, resulting in a temporary deformation or strain. Typically conducted with an ultrasound machine, images of the tissue are taken both before and after this deformation. The initial image serves as a reference, while the subsequent image illustrates how the tissue responds to the applied stress. The software connected to the imaging equipment calculates the displacement of tissue components between these two image sets. This displacement data is then employed to generate an elastogram, which is an image displayed in color-coded or grayscale format. The elastogram visualizes the relative elasticity or stiffness of the tissue, with stiffer regions typically represented in one color and more elastic areas in another [3]. However, strain elastography might be more suitable for superficial structures and not deeper organs, for example, liver, thyroid, and breast [4]. While most of the studies discuss liver fibrosis being quantified by elastography, the method is also being used to investigate other diseases—for example, pathologies of the rectum, appendix, pancreas, prostate, and other soft tissues [5,6,7].

It is important to note that elastography is still limited by operator dependent characteristics, which can influence many instrumental markers. For example, positive and negative predictive values and specificity and sensitivity [4]. Therefore, the primary objective of this review was to determine demographic characteristics and instrument-specific characteristics of various elastography modalities being used. The secondary objective was to determine if the instrument-specific values were dependent on the nature of the organs and type of modality being used.

## 2. Methodology

### 2.1. Protocol Development and Search Strategy

The protocol was developed in accordance with the Preferred Reporting Items for Systematic Reviews and Meta-Analyses (PRISMA). The review methods were established prior to the study. This study was conducted using PICO (patient, intervention, comparison, and outcomes) strategy for our research question. The keywords used in the search strategy are as described in Supplementary Table S1.

### 2.2. Data Extraction

We screened PubMed and Web of Science for conducting our search using Boolean operators (“OR”, “AND”) for the MESH terms described in Supplementary Table S1. This systematic review included articles published in English during the last fifteen years that fulfilled the following inclusion criteria: (1) articles discussing ultrasound elastography, shear wave elastography or strain elastography; (2) articles specifically documenting optimal sensitivity, specificity, area under the curve, positive and negative likelihood ratio, positive predictive value, and negative predictive values. Using the PICO strategy, the patients who had been evaluated using ultrasound elastography, shear wave elastography or strain elastography were included. The interventions in this regard were the types of elastography used (ultrasound elastography, shear wave elastography or strain elastography). The outcome variables were characteristics such as optimal sensitivity, specificity, area under the curve, positive and negative likelihood ratio, positive predictive value, and negative predictive values. These outcomes were compared to the patients with non-gastrointestinal pathologies. The systematic review included observational studies and randomized-controlled trials to minimize population bias and improve measurement of qualitative variables. During the initial search, 321 articles were found on PubMed and 40 articles on Web of Science. Duplicates were deleted after the first search. Two independent reviewers screened the remaining studies based on inclusion criteria and reviewed the abstracts initially progressing to full-text articles if criteria were met. Zotero and Rayyan applications were utilized. A third reviewer was called in to resolve any potential disagreements.

**Figure 1:**
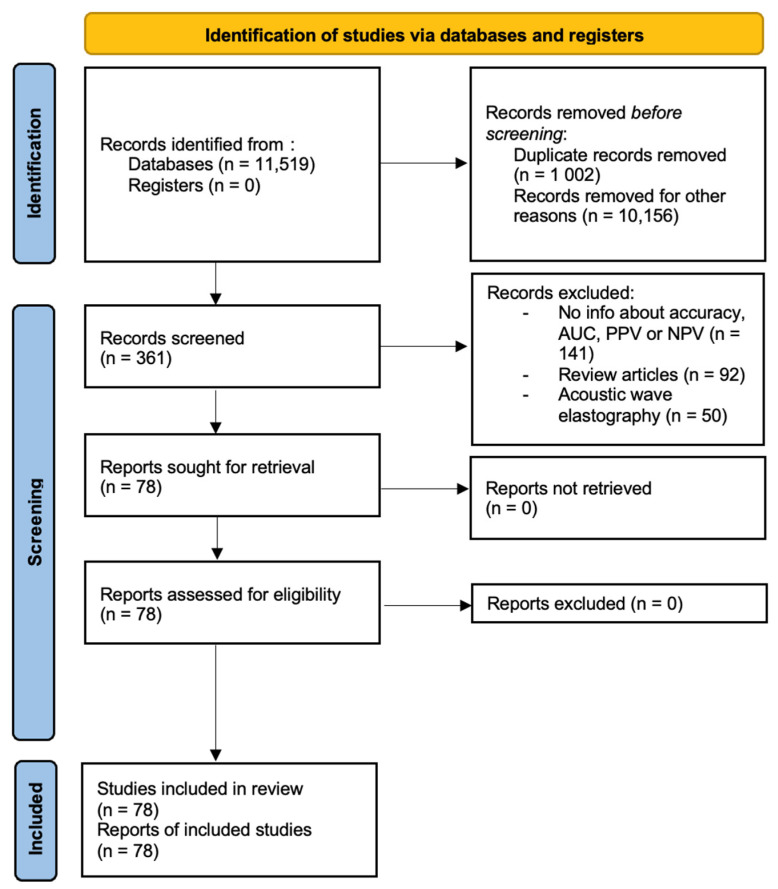
PRISMA diagram.

Information about study design, year, location where study was conducted, published journal, demographic data, diagnostic modalities, pathologies, and modality-specific values were extracted from the records by one reviewer. The extracted data was double-checked for accuracy and completeness. Studies were removed from the analysis if translations of the articles were not available, copies of complete articles were not available or if information about device-specific characteristics was complete. Papers that had not been peer-reviewed were also excluded. The preliminary data was recorded in an excel spreadsheet and final analysis contained 78 studies. This has been presented in Figure 1. 

### 2.3. Risk of Bias

The quality of the studies was assessed by tools published by Cochrane Reviews [8] [Table 1, Supplementary Table S2]

Statistical analysis was conducted through Statistical Package for the Social Sciences (SPSS) version 21 (IBM Corp., Armonk, NY, USA). Mean ± standard deviation was used for continuous variables. Frequency or percentages were used for qualitative variables. Statistical tests including chi-square, independent *t*-test, and analysis of variance were utilized. Logistic regression models were used to determine predictors for multiple outcomes. A *p*-value < 0.05 was considered significant.

## 3. Results

### 3.1. Demographic Characteristics

The review included 78 articles: 69 prospective studies, 7 retrospective studies, and 2 randomized controlled trials. The total number of cases included in the review was 13,689. Data regarding gender was available for 8787 cases. The majority of the cases were male (50.50%). Information about age was available for 12,753 cases. The mean age of the study population was 42.73 ± 4.41 years. The data extraction for the articles has been mentioned in Table 2 [9,10,11,12,13,14,15,16,17,18,19,20,21,22,23,24,25,26,27,28,29,30,31,32,33,34,35,36,37,38,39,40,41,42,43,44,45,46,47,48,49,50,51,52,53,54,55,56,57,58,59,60,61,62,63,64,65,66,67,68,69,70,71,72,73,74,75,76,77,78,79,80,81,82,83,84,85,86]. 

The types of elastography techniques utilized have been discussed in Supplementary Table S3 and the sources of funding have been discussed in Supplementary Table S4. 

The lowest positive predictive value of elastography was observed by Casariego et al., estimated to be 8.3% for real-time ultrasound elastography [9]. However, relatively higher positive predictive values were observed by Yoon et al. and Lin et al. [18,71]. The negative predictive values of elastography were relatively similar, ranging from 70% to 100%. However, lower negative predictive values were noted for shear wave elastography by Sporea et al. [28]. The lowest sensitivity was observed by Casariego et al., estimated to be 25% for real-time ultrasound elastography [9]. The lowest specificity was noted by Verhoeven et al. for strain elastography [9]. 

### 3.2. Imaging Modalities and Their Utility

Shear wave elastography was most involved (38/78; 48.7%), followed by real-time ultrasound elastography (26/78; 33.3%) and strain elastography (14/78; 17.9%). Organs involved in gastroenterology-based diseases were investigated in 34/78 cases (45.3%), of which liver (21/78; 34.6%) was the most common, followed by the pancreas (10/78; 12.8%). Other common organs that were investigated include the thyroid (21.8%) and lymph nodes (29.5%). Elastography was commonly used in diagnosis of malignancy for lymph nodes (26.3%), thyroid (18.8%), pancreas (5.0%), and prostate (2.5%) and for diagnostic accuracy in liver fibrosis (15.0%) and thyroid (11.3%). 

### 3.3. Subgroup Analyses

The subgroup analysis for different organs is shown in Table 3.

Real-time ultrasound elastography was commonly used for lymph nodes. Shear wave elastography was commonly used for liver. Strain elastography was also used for both pancreas and lymph nodes. However, the specificity of the elastography was greatest for the appendix. Similar results were noted for sensitivities; higher sensitivities were observed for the rectum, pancreas, and appendix. Higher accuracies were also observed for rectum. Additionally, higher positive predictive values were noted for elastography focused on appendix and rectum. 

Table 4 discusses the applications of elastography in organs. 

Modalities utilized were significantly associated with organ-based pathologies (*p* < 0.05). Although data for accuracy was limited as seen in Table 4, higher values were observed for the pancreas (*p* < 0.05). In the cases of the esophagus and rectum, the elastography modalities were associated with endoscopic ultrasound. The study focused on soft tissue and discussed the importance of elastography in patellar tendons. The different measures of all elastography modalities were used to determine indicators of better performance via multivariate regression analysis as shown in Table 5 and Table 6.

Elastography for gastrointestinal organs was associated with higher sensitivity, positive predictive value, and negative predictive values (*p* < 0.05) but lower specificity (*p* < 0.05). Additionally, real-time ultrasound elastography was associated with increased specificity, accuracy, and positive and negative predictive values (*p* < 0.05). 

## 4. Discussion

This is the first study to provide an overview of elastography in gastrointestinal organs. About 50.5% of the study population was male (50.5%), and the mean age of the participants was 42.73 ± 4.41 years. Unalp-Arida et al. discussed the application of elastography in liver stiffness and observed a comparatively lower proportion of males in the study population and an older age group [87]. The shear wave technique was commonly involved in elastography, particularly for the liver [29,30,31,32,33,34,35,36,37,38,39,40,41,42,43]. While shear wave technique might be hampered by inflammation, cholestasis, and congestion, strain technique remains relatively unaffected, increasing the diagnostic yield of elastography [88]. Furthermore, higher estimates of positive predictive value and accuracy were noted for strain elastography for the liver. This is consistent with results from a previous study [89]. The same mechanism can be attributed to the use in lymph nodes. 

Higher accuracy was associated with pancreatic imaging (*p* < 0.05). This finding was different compared to a previous study [90]. Usually, elastography has been considered difficult to perform due to the size of the pancreas [90]. However, strain elastography has an advantage in this regard because an additional static force is present in addition to aortic pulsations allowing for better study of the pancreatic body. However, the use in the pancreatic head and tail might still be limited [90]. 

Elastography involving gastrointestinal organs were also associated with higher sensitivity, positive predictive value, and negative predictive values (*p* <0.05) but lower specificity (*p* < 0.05). Apart from pancreatic diseases, rectal diseases also add to the increased likelihood of these values. Tissue stiffness is usually increased in malignancy and therefore, rectal malignancies of advanced stages would be better diagnosed using elastography as seen in the studies discussed [89]. Additionally, real-time ultrasound elastography was associated with increased specificity, accuracy, and positive and negative predictive values (*p* < 0.05). These findings were discussed by another study on breast disease, in which increased specificity, accuracy, and positive and negative predictive values were observed for breast lesions in cases of real-time ultrasound elastography [91]. 

### 4.1. Use in Non-Gastrointestinal Tissues

Elastography is widely employed in non-gastrointestinal organs due to its distinctive characteristics. It is particularly useful in the evaluation of thyroid nodules. Evaluation of small nodules is limited because of increased distance from the transducer and in patients with larger neck circumference. The index of compression on elastography can help in determining both the size and nature of the nodule [11]. The varying tissue elasticity is also of immense importance in the evaluation of lymph nodes. Altered tissue composition represented by a higher tissue stiffness is more suggestive of malignancy. Malignant lesions are usually suspected in cases of excessive keratin deposition or microcalcifications that hamper stiffness index; these features are more suggestive of cortical damage [47]. 

Desmoplastic reaction might be limited in cases of non-Hodgkin lymphoma and require the use of shear-wave elastography [47]. In a few cases, the combined use of elastography with other techniques can allow preoperative assessment of axillary lymph nodes in patients with breast cancer [57]. The technique can be optimized via the use of various reference points—for example, the carotid artery and neck muscles in patients with thyroid cancer have good sensitivity and better negative predictive values for determining benign and malignant natures of thyroid nodules [86]. 

Shear wave elastography is more utilized in cases of prostatic malignancies. Calcifications on shear wave elastography might present false positive results, but extracapsular extension of malignancies can be better visualized [73]. 

### 4.2. Use in Gastrointestinal Tissues

Elastography is also being used in many gastrointestinal diseases, specifically the liver and pancreas. Transient elastography is an accurate predictor of liver fibrosis and provides an important correlation with the recurrence of viral illnesses, including hepatitis C [27,36]. Perisinusoidal fibrosis was observed to influence the results of transient elastography in this set of patients leading to a better correlation with recurrence of disease [27]. The varied forms of elastography, including Fibro Scan, can also provide insight to hepatic steatosis, leading to better diagnosis of nonalcoholic fatty liver disease in morbidly obese patients [30]. Other forms of chronic liver disease can also be evaluated, including cirrhotic liver disease and portal hypertensive gastropathy [24,25].

Rectal lesions have also been assessed using elastography for preoperative assessment [74]. Pancreatic lesions have also been commonly assessed with strain elastography. A few measures have been discussed in this regard. Malignant lesions usually have a significantly higher lesion-to-parenchyma strain ratio and lesion-to-wall ratio [78]. The technique does not increase operation time or risk of adverse effects on evaluation [78]. Endoscopic ultrasound elastography has also been used in the identification of pancreatic duct dilatation that correlated with higher stiffness index and head-based locations in patients with pancreatic cancer [77]. The elastic modulus as shown by elastography is also useful in appendicitis and other infections. However, there are a few limitations in the evaluation of the appendix. The signal can be displaced by an anteriorly located cecum in the retrocecal appendix [72]. Most importantly, because of the location of the appendix, the use of shear wave elastography might be limited because the shear waves decrease in intensity during propagation [72].

The esophagus is also being evaluated using elastography. Reflux esophagitis is caused by acid retention or reflux in the lower esophagus. The esophagogastric junction prevents reflux and has varying pressure depending on the contraction of the lower esophageal sphincter [75]. The change in stiffness with appropriate reference points—for example, the liver—correlated with motility of the esophagogastric junction. Therefore, a greater change in stiffness indirectly implied normal movements of the esophagogastric junction [75]. Similar underlying principles have been used in esophageal cancer for evaluation of lymph nodes [53]. 

#### Limitations of the Study

The main strength of the study is the comparison of measures in various organs and pathologies using elastography on a large scale. There are a few limitations to the study. There was limited availability of data regarding accuracy. Additionally, multiple studies used qualitative measures that could not be compared in a pair-wise fashion despite producing significant results in the studies discussed. 

## 5. Conclusions

Ultrasound-based elastography is gradually becoming a widely used source in many diseases. However, the use is still more common and apparently accurate in gastrointestinal diseases. Shear wave elastography was commonly used for the liver, and strain elastography was commonly utilized for the pancreas. However, accuracy and positive predictive value of strain elastography in the liver would help in navigating through differential diagnoses of various pathologies. Elastography techniques might help in minimizing the impact of inflammation in visualizing lesions in both gastrointestinal and non-gastrointestinal tissues, for example, prostate. The addition of different indices—for example, motility index in the esophagus—can help to diagnose risk of reflux early. The utility and outcomes are, however, dependent on a few instrument-specific characteristics and operator-dependent characteristics—for example, feasibility of use and experience of the operator. Furthermore, the addition of real-time imaging and determination of appropriate cutoff values for optimal results are other factors that must be considered. Despite its promising utility as a tool, further studies are needed to determine the associated factors and optimal output for both gastrointestinal and non-gastrointestinal diseases. Many future applications of elastography are yet to be investigated including cancer detection and monitoring and use in minimally invasive procedures. With the promising advent of artificial intelligence, further studies using both artificial intelligence and elastography might present a new era of interventions.

## Figures and Tables

**Table 1:** Quality Assessment.

Judgement	Percentage %	Number	Clinical Trials (*n* = 2)	Prospective Studies (*n* = 70)	Retrospective Studies (*n* = 6)
Good	85.9	67	2	62	3
Fair	14.1	11	0	8	3
Poor	0	0	0	0	0

**Table 2:** Findings of the studies discussed.

Author	*N*	Age (years)	Males	Females	Sensitivity	Specificity	AUC	Accuracy	PPV	NPV
Casariego et al. [9]	128	56.1	12	116	25	86.9	-	-	8.3	96.1
Azizi et al. [10]	676	51.2	97	579	79.3	71.5	-	-	26.8	96.3
Dighe et al. [11]	35	51.6	7	28	100	60	0.81	-	-	-
Unlütürk et al. [12]	194	47	37	157	47	80	-	72	44	83
Cakal et al. [13]	224	46.5	26	198	79.4	98.1	0.89	-	-	-
Asteria et al. [14]	66	55	54	12	94.1	81	-	83.7	55.2	98.2
Elsayed et al. [15]	88	45	14	74	75	69.8	-	70.8	38.7	91.6
Cantisiani et al. [16]	50	58	4	46	90	92.7	0.96	86.9	-	-
Du et al. [17]	142	40	58	122	94.4	87.1	-	70.4	65.9	79.2
Yoon et al. [18]	169	50.3	31	138	81	56.5	0.69	65.5	52	83.6
Yang et al. [19]	205	50.25	38	167	100	-	-	94.8	-	-
Gay et al. [20]	81	59.4	24	57	50	86.7	0.73	-	-	-
Russ et al. [21]	3543	54	0	0	98.5	44.7	-	48.3	-	99.8
Li et al. [22]	280	48	64	216	76.5	78.4	0.83	-	-	-
Wu et al. [23]	19	46	7	12	16.7	100	-	88	-	-
Seong et al. [24]	196	51.1	35	161	50	57.2	-	56.3	14	89.2
Bhatia et al. [25]	74	52.8	16	58	76.9	71.1	-	-	-	-
Huang et al. [26]	69	44	17	52	68.75	91.3	0.84	-	-	-
Rigamonti et al. [27]	90	58	73	17	93	93	0.9	-	74	99
Sporea et al. [28]	199	49.79	61	138	59.6	93.3	0.77	-	98	30.1
Abrams et al. [29]	43	0	0	0	69.2	73.3	-	-	52.9	84.6
Garg et al. [30]	76	39.3	18	58	63.6	87.7	0.83	-	43	93
Ramirez et al. [31]	85	45.4	65	20	100	27.7	-	60	52.5	100
Malik et al. [32]	404	53	283	121	92	88	0.9	-	87	90
Corpechot et al. [33]	73	40.7	59	27	94	87	0.91	88	53	99
Miailhes et al. [34]	59	43	49	10	92	94	-	93	79	98
Lee et al. [35]	280	43	194	86	72	65	0.75	-	27	93
Harada et al. [36]	56	63.1	30	26	100	98	0.99	-	83	100
Gara et al. [37]	109	52	5	104	90	78	0.91		62	95
Pang et al. [38]	2052	51	1134	918	41	93	-	90	20	97
Dominguez et al. [39]	80	56	26	64	88	98	0.86	-	88	98
Seo et al. [40]	381	44.1	251	130	76.6	80.3	0.83	-	-	-
Xie et al. [41]	160	52.7	134	26	77	80	0.83	-	59	90
Beckebaum et al. [42]	157	52.5	44	113	95.8	75	-	85.4	94.7	79.3
Obara et al. [43]	114	56	55	59	90	84	0.94	-	71	96
Endo et al. [44]	189	62	95	94	81.5	86	0.90	-	-	-
Ooi et al. [45]	35	22.2	15	20	82.5	33.3	-	61.4	66.7	57.1
Jin et al. [46]	119	0	34	85	88	95	-	94	54	99
Chae et al. [47]	62	0	0	0	84	75	0.82	79.6	83.3	73.7
Desmots et al. [48]	56	49	31	25	87	88	0.90	-	87	88
Alam et al. [49]	37	0	0	0	83	100	-	89	-	-
Lo et al. [50]	109	53	54	55	83.3	64.7	-	68.8	40	93.2
Lenghel et al. [51]	70	0	0	0	64.29	94	0.85	76.7	93.8	65.3
Chang et al. [52]	140	55.3	2	138	60.26	96.77	-	76.4	95.9	66.4
Paterson et al. [53]	48	67	38	10	83	96	-	90	95	86
Choi et al. [54]	62	0	0	0	80.7	66.7	-	73.4	69.4	78.6
Choi et al. [55]	15	0	0	0	91.2	97	-	94	96.9	91.4
Fujiwara et al. [56]	122	68.4	94	28	72.1	84	-	79.7	72.1	84
Verhoeven et al. [57]	327	66	200	127	98	22	0.77	58	54	91
Ng et al. [58]	107	58	0	0	96	56.1	0.81	74.7	65.7	94.1
Seo et al. [59]	54	0	0	0	76.47	100	0.88	-	100	71.43
Ogata et al. [60]	20	0	0	0	92	100	-	95	-	-
Taylor et al. [61]	50	57	0	50	100	48	-	-	58	100
Acu et al. [62]	168	37.1	86	82	71.6	76.5	-	75	-	-
Fournier et al. [63]	116	60.2	80	34	87	68	-	-	80	77
Korrungruang et al. [64]	72	58.3	41	31	100	70.8	0.85	-	93.2	100
Larsen et al. [65]	56	0	0	0	59	82	-	73	68	76
Harve et al. [66]	61	0	0	0	65	62.5	-	-	45.8	78.5
Che et al. [67]	81	46.6	0	0	91.1	83.3	0.93	87.7	-	-
Pehlivan et al. [68]	23	56.43	0	0	82.4	84.6	-	83.3	87	78
Fang et al. [69]	42	59.57	30	12	93.33	89.36	0.96	90.3	97.6	73.7
Sun et al. [70]	56	56.07	32	24	88.57	100	-	94.1	100	89.2
Lin et al. [71]	94	62.8	65	29	90.6	82.6	-	85.2	71.6	94.7
Cha et al. [72]	52	36	28	24	93	100	-	-	100	85
Barr et al. [73]	53	64.2	0	0	96.2	96.2	-	-	69.4	99.6
Li et al. [74]	96	59	55	41	93	88.3	-	90.6	94.4	80
Suhara et al. [75]	108	66	56	52	92.7	65.6	0.84	74.5	-	-
Rustemović et al. [76]	149	63	73	76	100	95	-	-	92	100
Kataoka et al. [77]	126	70	116	10	95	53	-	-	68	91
Carrara et al. [78]	100	0	0	0	88.4	78.8	0.87	-	76.7	86.7
Ignes et al. [79]	218	0	0	0	84	67	-	-	56	89
Okasha et al. [80]	172	55.7	120	52	99	63	-	88	87	96
Ahmad et al. [81]	11	0	0	0	93	93	-	-	98	81
Aghaghazvini et al. [82]	117	52.98	56	61	90	77.67	0.91	-	42	98
Wang et al. [83]	185	45	0	0	93.8	50	-	86.1	89.7	63.3
Azizi et al. [84]	71	0	0	0	66.3	87.8	-	-	36.1	96.2
Wang et al. [85]	445	44.1	0	0	94.3	53.3	0.74	80.5	79.8	82.8
Kratky et al. [86]	61	50	14	47	67	89	-	-	78	83

AUC: area under the curve, PPV: positive predictive value, NPV: negative predictive value.

**Table 3:** Subgroup analysis for different organs.

Variable	Thyroid	Prostate	Liver	Soft Tissue	Lymph Node	Breast	Esophagus	Appendix	Rectum	Pancreas	*p*-Value
RUE	6	0	1	1	12	1	1	0	0	4	0.02
SWE	7	1	19	0	7	0	0	1	1	2	
SE	3	0	1	0	4	2	0	0	0	3	
SWE + SE	1	0	0	0	0	0	0	0	0	0	
Spec	73.58 ± 22.39	94.60 ± 2.26	82.75 ± 15.98	33.30 ± 22.92	78.39 ± 19.35	87.82 ± 18.36	80.80 ± 21.50	93.00 ± 22.92	83.30 ± 2.92	91.36 ± 16.13	0.346
Sens	75.08 ± 22.92	94.60 ± 2.26	82.38 ± 15.58	82.50 ± 22.92	85.26 ± 11.90	72.48 ± 10.79	87.85 ± 6.86	100.00 ± 32.90	98.00 ± 22.12	93.28 ± 6.91	0.165
AUC	0.31 ± 0.21	N/A	0.65 ± 0.40	N/A	0.31 ± 0.21	0.29 ± 0.11	0.42 ± 0.30	N/A	N/A	0.17 ± 0.03	>0.05
Acc	41.56 ± 40.39	N/A	21.92 ± 19.20	61.40 ± 19.92	59.28 ± 38.16	49.94 ± 43.28	82.25 ± 10.16	N/A	90.60 ± 12.96	17.6 ± 9.35	0.002
PPV	28.52 ± 20.56	83.70 ± 20.22	54.95 ± 32.34	66.70 ± 2.92	61.98 ± 34.12	88.44 ± 16.61	47.50 ± 27.18	100.00 ± 42.66	94.40 ± 11.95	75.94 ± 14.50	0.66
NPV	55.80 ± 44.86	90.30 ± 13.15	75.89 ± 37.17	57.1 ± 32.34	70.33 ± 35.23	72.15 ± 6.12	43.00 ± 20.81	85.00 ± 32.34	80.00 ± 32.90	9.25 ± 5.40	>0.05

RUE: real-time ultrasound elastography, SWE: shear wave elastography, SE: strain elastography, Spec: specificity, Sens: sensitivity, Acc: Accuracy, N/A: Not Applicable.

**Table 4:** Applications in Various Organs.

Organs	Applications
Thyroid	Diagnostic Accuracy in Malignancy (15/78; 19.2%)
	Diagnostic Accuracy in Cystic Disease (2/78; 2.6%)
Liver	Diagnostic Accuracy in Liver Fibrosis (15/78; 19.2%)
	Prognosis in Liver Fibrosis (2/78; 2.6%)
	Diagnostic Accuracy in Cirrhosis (2/78; 2.6%)
	Diagnostic Accuracy in Primary Sclerosing Cholangitis (1/78; 1.3%)
	Diagnostic Accuracy in Hepatocellular Carcinoma (1/78; 1.3%)
Lymph Node	Diagnostic Accuracy in Lymph Node Malignancy (23/78; 29.5%)
Prostate	Diagnosis in Prostatic Malignancy (1/78; 1.3%)
Breast	Diagnosis in Cystic Disease (2/78; 2.6%)
	Diagnosis in Breast Malignancy (1/78; 1.3%)
Appendix	Diagnosis in Appendicitis (1/78; 1.3%)
Esophagus	Diagnosis of Esophagitis (1/78; 1.3%)
Rectum	Diagnosis of Rectal Malignancy (1/78; 1.3%)
Pancreas	Diagnosis of Pancreatic Malignancy (9/78;11.5%)
Patellar Tendon	Diagnosis (1/78; 1.3%)

**Table 5:** Measures in gastrointestinal organs.

Measures	Adjusted Odds Ratio	95% Confidence Interval	*p*-Value
Sensitivity	1.70	1.37–2.13	0.00
Specificity	0.43	0.34–0.55	0.00
Accuracy	0.66	0.00–0.75	>0.05
Positive Predictive Value	1.94	1.52–2.48	0.00
Negative Predictive Value	3.80	2.95–4.90	0.00

**Table 6:** Measures in real-time ultrasound elastography.

Measures	Adjusted Odds Ratio	95% Confidence Interval	*p*-Value
Sensitivity	1.06	0.58–1.44	0.52
Specificity	1.45	1.29–1.64	0.00
Accuracy	25.15	24.90–25.40	0.00
Positive Predictive Value	23.86	23.70–24.02	0.00
Negative Predictive Value	24.41	24.27–29.36	0.00

## Data Availability

Data can be made available by special request addressed to the corresponding author.

## Supplementary Materials

The following supporting information can be downloaded at: https://www.mdpi.com/article/10.3390/diagnostics13213302/s1, Supplementary Table S1 details the MESH terms used in the search. Supplementary Table S2 discusses the risk of bias of the studies included. Supplementary Table S3 includes the types of elastography techniques used. Supplementary Table S4 includes the sources of funding of all the included studies. Supplementary Table S5 represents the PRISMA checklist.
