# Supplementary material for: Associations of Real-Time Ultrasound and Strain and Shear Wave Elastography with Gastrointestinal Organs: A Systematic Review
Source: Diagnostics (Basel). 2023 Oct 25;13(21):3302. doi: 10.3390/diagnostics13213302 (PMC10649379; doi:10.3390/diagnostics13213302)
Supplement: Supplementary file 1 [file diagnostics-13-03302-s001.zip › diagnostics-2643689-supplementary.pdf]

Supplementary Table S1

(ultrasound elastography[MeSH Terms]) AND (colon[MeSH Terms]) "(""diagnostic imaging""[MeSH Subheading] OR (""diagnostic""[All Fields] AND ""imaging""[All Fields]) OR ""diagnostic imaging""[All Fields] OR ""ultrasound""[All Fields] OR ""ultrasonography""[MeSH Terms] OR ""ultrasonography""[All Fields] OR ""ultrasonics""[MeSH Terms] OR ""ultrasonics""[All Fields] OR ""ultrasounds""[All Fields] OR ""ultrasound s""[All Fields]) AND ""elasticity imaging techniques""[MeSH Terms] AND ""colon""[MeSH Terms]"
(ultrasound elastography[MeSH Terms]) AND (rectum[MeSH Terms]) "(""diagnostic imaging""[MeSH Subheading] OR (""diagnostic""[All Fields] AND ""imaging""[All Fields]) OR ""diagnostic imaging""[All Fields] OR ""ultrasound""[All Fields] OR ""ultrasonography""[MeSH Terms] OR ""ultrasonography""[All Fields] OR ""ultrasonics""[MeSH Terms] OR ""ultrasonics""[All Fields] OR ""ultrasounds""[All Fields] OR ""ultrasound s""[All Fields]) AND ""elasticity imaging techniques""[MeSH Terms] AND ""rectum""[MeSH Terms]"
(ultrasound elastography[MeSH Terms]) AND (pancreas[MeSH Terms]) "(""diagnostic imaging""[MeSH Subheading] OR (""diagnostic""[All Fields] AND ""imaging""[All Fields]) OR ""diagnostic imaging""[All Fields] OR ""ultrasound""[All Fields] OR ""ultrasonography""[MeSH Terms] OR ""ultrasonography""[All Fields] OR ""ultrasonics""[MeSH Terms] OR ""ultrasonics""[All Fields] OR ""ultrasounds""[All Fields] OR ""ultrasound s""[All Fields]) AND ""elasticity imaging techniques""[MeSH Terms] AND ""pancreas""[MeSH Terms]"
(ultrasound elastography[MeSH Terms]) AND (soft tissue[MeSH Terms]) "(""diagnostic imaging""[MeSH Subheading] OR (""diagnostic""[All Fields] AND ""imaging""[All Fields]) OR ""diagnostic imaging""[All Fields] OR ""ultrasound""[All Fields] OR ""ultrasonography""[MeSH Terms] OR ""ultrasonography""[All Fields] OR ""ultrasonics""[MeSH Terms] OR ""ultrasonics""[All Fields] OR ""ultrasounds""[All Fields] OR ""ultrasound s""[All Fields]) AND ""elasticity imaging techniques""[MeSH Terms] AND (""soft""[All Fields] AND ""tissues""[MeSH Terms])"
(ultrasound elastography[MeSH Terms]) AND (intestine[MeSH Terms]) "(""diagnostic imaging""[MeSH Subheading] OR (""diagnostic""[All Fields] AND ""imaging""[All Fields]) OR ""diagnostic imaging""[All Fields] OR ""ultrasound""[All Fields] OR ""ultrasonography""[MeSH Terms] OR ""ultrasonography""[All Fields] OR ""ultrasonics""[MeSH Terms] OR ""ultrasonics""[All Fields] OR ""ultrasounds""[All Fields] OR ""ultrasound s""[All Fields]) AND ""elasticity imaging techniques""[MeSH Terms] AND ""intestines""[MeSH Terms]"

(ultrasound elastography[MeSH Terms]) AND (liver[MeSH Terms]) "(""diagnostic imaging""[MeSH Subheading] OR (""diagnostic""[All Fields] AND ""imaging""[All Fields]) OR ""diagnostic imaging""[All Fields] OR ""ultrasound""[All Fields] OR ""ultrasonography""[MeSH Terms] OR ""ultrasonography""[All Fields] OR ""ultrasonics""[MeSH Terms] OR ""ultrasonics""[All Fields] OR ""ultrasounds""[All Fields] OR ""ultrasound s""[All Fields]) AND ""elasticity imaging techniques""[MeSH Terms] AND ""liver""[MeSH Terms]" "
(ultrasound elastography[MeSH Terms]) AND (stomach[MeSH Terms]) "(""diagnostic imaging""[MeSH Subheading] OR (""diagnostic""[All Fields] AND ""imaging""[All Fields]) OR ""diagnostic imaging""[All Fields] OR ""ultrasound""[All Fields] OR ""ultrasonography""[MeSH Terms] OR ""ultrasonography""[All Fields] OR ""ultrasonics""[MeSH Terms] OR ""ultrasonics""[All Fields] OR ""ultrasounds""[All Fields] OR ""ultrasound s""[All Fields]) AND ""elasticity imaging techniques""[MeSH Terms] AND ""stomach""[MeSH Terms]"
(ultrasound elastography[MeSH Terms]) AND (lymph nodes[MeSH Terms]) "(""diagnostic imaging""[MeSH Subheading] OR (""diagnostic""[All Fields] AND ""imaging""[All Fields]) OR ""diagnostic imaging""[All Fields] OR ""ultrasound""[All Fields] OR ""ultrasonography""[MeSH Terms] OR ""ultrasonography""[All Fields] OR ""ultrasonics""[MeSH Terms] OR ""ultrasonics""[All Fields] OR ""ultrasounds""[All Fields] OR ""ultrasound s""[All Fields]) AND ""elasticity imaging techniques""[MeSH Terms] AND ""lymph nodes""[MeSH Terms]"
37 (ultrasound elastography[MeSH Terms]) AND (cardiac[MeSH Terms]) "(""diagnostic imaging""[MeSH Subheading] OR (""diagnostic""[All Fields] AND ""imaging""[All Fields]) OR ""diagnostic imaging""[All Fields] OR ""ultrasound""[All Fields] OR ""ultrasonography""[MeSH Terms] OR ""ultrasonography""[All Fields] OR ""ultrasonics""[MeSH Terms] OR ""ultrasonics""[All Fields] OR ""ultrasounds""[All Fields] OR ""ultrasound s""[All Fields]) AND ""elasticity imaging techniques""[MeSH Terms] AND ""heart""[MeSH Terms]"
(ultrasound elastography[MeSH Terms]) AND (lungs[MeSH Terms]) "(""diagnostic imaging""[MeSH Subheading] OR (""diagnostic""[All Fields] AND ""imaging""[All Fields]) OR ""diagnostic imaging""[All Fields] OR ""ultrasound""[All Fields] OR ""ultrasonography""[MeSH Terms] OR ""ultrasonography""[All Fields] OR ""ultrasonics""[MeSH Terms] OR ""ultrasonics""[All Fields] OR ""ultrasounds""[All Fields] OR ""ultrasound s""[All Fields]) AND ""elasticity imaging techniques""[MeSH Terms] AND ""lung""[MeSH Terms]"
(ultrasound elastography[MeSH Terms]) AND (thyroid[MeSH Terms]) "(""diagnostic imaging""[MeSH Subheading] OR (""diagnostic""[All Fields] AND ""imaging""[All Fields]) OR

""diagnostic imaging""[All Fields] OR ""ultrasound""[All Fields] OR ""ultrasonography""[MeSH Terms] OR ""ultrasonography""[All Fields] OR ""ultrasonics""[MeSH Terms] OR ""ultrasonics""[All Fields] OR ""ultrasounds""[All Fields] OR ""ultrasound s""[All Fields]) AND ""elasticity imaging techniques""[MeSH Terms] AND (""thyroid gland""[MeSH Terms] OR ""thyroid usp""[MeSH Terms])"

Supplementary Table S2: Risk of Bias in Studies

Author	Risk of Bias	Type of Study
Casariego et al [9]	Low risk	Clinical Trial
Azizi et al [10]	Some concerns	Prospective study
Dighe et al [11]	Some concerns	Prospective study
Unlütürk et al [12]	Low risk	Prospective study
Cakal et al [13]	Some concerns	Prospective study
Asteria et al [14]	Low risk	Prospective study
Elsayed et al [15]	Low risk	Prospective study
Cantisiani et al [16]	Low risk	Prospective study
Du et al [17]	Low risk	Prospective study
Yoon et al [18]	Low risk	Prospective study
Yang et al [19]	Some concerns	Retrospective Study
Gay et al [20]	Some concerns	Prospective study
Russ et al [21]	Low risk	Prospective study
Li et al [22]	Some concerns	Prospective study
Wu et al [23]	Some concerns	Retrospective Study
Seong et al [24]	Low risk	Retrospective Study
Bhatia et al [25]	Some concerns	Prospective study
Huang et al [26]	Some concerns	Prospective study
Rigamonti et al [27]	Low risk	Prospective study
Sporea et al [28]	Low risk	Prospective study
Abrams et al [29]	Some concerns	Prospective study
Garg et al [30]	Low risk	Prospective study
Ramirez et al [31]	Low risk	Prospective study
Malik et al [32]	Low risk	Prospective study
Corpechot et al [33]	Low risk	Prospective study

Miailhes et al [34]	Low risk	Prospective study
Lee et al [35]	Low risk	Prospective study
Harada et al [36]	Low risk	Prospective study
Gara et al [37]	Low risk	Prospective study
Pang et al [38]	Low risk	Prospective study
Dominguez et al [39]	Low risk	Prospective study
Seo et al [40]	Low risk	Prospective study
Xie et al [41]	Low risk	Prospective study
Beckebaum et al [42]	Low risk	Prospective study
Obara et al [43]	Low risk	Prospective study
Endo et al [44]	Some concerns	Prospective study
Ooi et al [45]	Some concerns	Prospective study
Jin et al [46]	Low risk	Prospective study
Chae et al [47]	Low risk	Prospective study
Desmots et al [48]	Low risk	Prospective study
Alam et al [49]	Low risk	Prospective study
Lo et al [50]	Some concerns	Prospective study
Lenghel et al [51]	Low risk	Prospective study
Chang et al [52]	Low risk	Prospective study
Paterson et al [53]	Low risk	Retrospective Study
Choi et al [54]	Low risk	Prospective study
Choi et al [55]	Low risk	Retrospective Study
Fujiwara et al [56]	Low risk	Clinical Trial
Verhoeven et al [57]	Low risk	Retrospective Study
Ng et al [58]	Low risk	Prospective Study
Seo et al [59]	Low risk	Prospective study
Ogata et al [60]	Low risk	Retrospective Study
Taylor et al [61]	Some concerns	Prospective study
Acu et al [62]	Low risk	Prospective study
Fournier et al [63]	Some concerns	Prospective study
Korrungruang et al [64]	Low risk	Prospective study
Larsen et al [65]	Low risk	Prospective study

Harve et al [66]	Low risk	Prospective study
Che et al [67]	Low risk	Prospective study
Pehlivan et al [68]	Low risk	Prospective study
Fang et al [69]	Low risk	Prospective study
Sun et al [70]	Low risk	Prospective study
Lin et al [71]	Low risk	Prospective study
Cha et al [72]	Low risk	Prospective study
Barr et al [73]	Low risk	Prospective study
Li et al [74]	Low risk	Prospective study
Suhara et al [75]	Low risk	Prospective study
Rustemović et al [76]	Low risk	Prospective study
Kataoka et al [77]	Low risk	Prospective study
Carrara et al [78]	Low risk	Prospective study
Ignes et al [79]	Low risk	Prospective study
Okasha et al [80]	Low risk	Prospective study
Ahmad et al [81]	Low risk	Prospective study
Aghaghazvini et al [82]	Low risk	Prospective study
Wang et al [83]	Low risk	Prospective study
Azizi et al [84]	Low risk	Prospective study
Wang et al [85]	Low risk	Prospective study
Kratky et al [86]	Low risk	Prospective study

Supplementary Table S3: Type of Elastography Investigated in Studies

Author	Elastography
Casariego et al [9]	Real-Time Ultrasound elastography
Azizi et al [10]	Shear wave elastography
Dighe et al [11]	Real-Time Ultrasound elastography
Unlütürk et al [12]	Real-Time Ultrasound elastography
Cakal et al [13]	Real-Time Ultrasound elastography
Asteria et al [14]	Real-Time Ultrasound elastography
Elsayed et al [15]	Real-Time Ultrasound elastography
Cantisiani et al [16]	Real-Time ultrasound elastography
Du et al [17]	Real-Time ultrasound elastography
Yoon et al [18]	Real-Time Ultrasound elastography
Yang et al [19]	Strain elastography
Gay et al [20]	Strain and shear wave elastography
Russ et al [21]	Real-Time Ultrasound elastography
Li et al [22]	Shear wave elastography
Wu et al [23]	Real-Time Ultrasound elastography
Seong et al [24]	Strain elastography
Bhatia et al [25]	Shear wave elastography
Huang et al [26]	Shear wave elastography
Rigamonti et al [27]	Shear wave elastography
Sporea et al [28]	Shear wave elastography
Abrams et al [29]	Shear wave elastography
Garg et al [30]	Shear wave elastography
Ramirez et al [31]	Shear wave elastography
Malik et al [32]	Shear wave elastography

Corpechot et al [33]	Shear wave elastography
Miailhes et al [34]	Shear wave elastography
Lee et al [35]	Shear wave elastography
Harada et al [36]	Shear wave elastography
Gara et al [37]	Shear wave elastography
Pang et al [38]	Shear wave elastography
Dominguez et al [39]	Shear wave elastography
Seo et al [40]	Shear wave elastography
Xie et al [41]	Shear wave elastography
Beckebaum et al [42]	Shear wave elastography
Obara et al [43]	Shear wave elastography
Endo et al [44]	Shear wave elastography
Ooi et al [45]	Real-time ultrasound elastography
Jin et al [46]	Strain elastography
Chae et al [47]	Shear wave elastography
Desmots et al [48]	Shear wave elastography
Alam et al [49]	Shear wave elastography
Lo et al [50]	Shear wave elastography
Lenghel et al [51]	Shear wave elastography
Chang et al [52]	Strain elastography
Paterson et al [53]	Real-time ultrasound elastography
Choi et al [54]	Strain elastography
Choi et al [55]	Shear wave elastography
Fujiwara et al [56]	Strain elastography
Verhoeven et al [57]	Strain elastography
Ng et al [58]	Shear wave elastography
Seo et al [59]	Shear wave elastography
Ogata et al [60]	Real-time elastography
Taylor et al [61]	Strain elastography
Acu et al [62]	Strain elastography
Fournier et al [63]	Strain elastography
Korrungruang et al [64]	Strain elastography

Larsen et al [65]	Real-time ultrasound elastography
Harve et al [66]	Strain elastography
Che et al [67]	Shear wave elastography
Pehlivan et al [68]	Real time elastography
Fang et al [69]	Real-time ultrasound elastography
Sun et al [70]	Real-time ultrasound elastography
Lin et al [71]	Real-time ultrasound elastography
Cha et al [72]	Shear wave elastography
Barr et al [73]	Shear wave elastography
Li et al [74]	Shear wave elastography
Suhara et al [75]	Real-time elastography
Rustemović et al [76]	Real-time elastography
Kataoka et al [77]	Real-time ultrasound elastography
Carrara et al [78]	Real-time ultrasound elastography
Ignes et al [79]	Real-time ultrasound elastography
Okasha et al [80]	Real-time ultrasound elastography
Ahmad et al [81]	Shear wave elastography
Aghaghazvini et al [82]	Shear wave elastography
Wang et al [83]	Shear wave elastography
Azizi et al [84]	Real-Time elastography
Wang et al [85]	Shear wave elastography
Kratky et al [86]	Strain elastography

Supplementary Table S4: Sources of Funding of studies discussed

Author	Sources of Funding
Casariego et al [9]	No sources received
Azizi et al [10]	No sources declared
Dighe et al [11]	No sources declared
Unlütürk et al [12]	No sources received
Cakal et al [13]	No sources declared
Asteria et al [14]	No sources declared
Elsayed et al [15]	No sources received
Cantisiani et al [16]	No sources received
Du et al [17]	Funding received from Central Hospital of Shanghai Putuo District Yuying talent project
Yoon et al [18]	No sources declared
Yang et al [19]	No sources declared
Gay et al [20]	No sources declared
Russ et al [21]	No sources received
Li et al [22]	No sources declared
Wu et al [23]	Funding received from Planned Project of Jiangsu Province Health Department and Clinical Research grant of Wujieping Medical Foundation.
Seong et al [24]	No sources declared
Bhatia et al [25]	No sources received
Huang et al [26]	No sources received

Rigamonti et al [27]	Funding received from Roche S.P.A., Italy and Mr Aldo Antognozzi and Consorzio Trapianti d'Organo.
Sporea et al [28]	No sources received
Abrams et al [29]	No sources received
Garg et al [30]	No sources received
Ramirez et al [31]	No sources declared
Malik et al [32]	No sources received
Corpechot et al [33]	Funding from Fonds CSP
Miailhes et al [34]	No sources received
Lee et al [35]	Funding received from Korea Health 21 R&D project, Ministry of Health, Welfare and Family Affairs, Republic of Korea (No. A050021).
Harada et al [36]	No sources received
Gara et al [37]	No sources received
Pang et al [38]	No sources received
Dominguez et al [39]	Funding received in part from Fundación de Investigación Mutua Madrileña
Seo et al [40]	No sources received
Xie et al [41]	Funding received from National Natural Science Foundation of China (81770607 and 81772626).
Beckebaum et al [42]	No sources received
Obara et al [43]	No sources received
Endo et al [44]	No sources received
Ooi et al [45]	No sources received
Jin et al [46]	No sources received
Chae et al [47]	No sources received
Desmots et al [48]	No sources received
Alam et al [49]	No sources received

Lo et al [50]	No sources received
Lenghel et al [51]	No sources received
Chang et al [52]	Funding received from National Natural Science Foundation of China (grants 81470079 and 81172078) and the Key Project of the Shanghai Municipal Health Bureau (grant 201440038).
Paterson et al [53]	No sources received
Choi et al [54]	No sources received
Choi et al [55]	No sources received
Fujiwara et al [56]	Honoraria and lecture fees from Olympus Medical Systems for EBUS-TBNA training courses
Verhoeven et al [57]	No sources received
Ng et al [58]	Funding received from University of Malaya Faculty Research Grant (RU Grant-Faculty Programme (Project No: GPF06C-2018), Fundamental Research Grant Scheme (FRGS/1/2019/SKK03/UM/01/1)
Seo et al [59]	No sources received
Ogata et al [60]	No sources received
Taylor et al [61]	No sources received
Acu et al [62]	No sources received
Fournier et al [63]	No sources received
Korrungruang et al [64]	No sources received
Larsen et al [65]	No sources received
Harve et al [66]	No sources received
Che et al [67]	No sources received
Pehlivan et al [68]	No sources received
Fang et al [69]	No sources received
Sun et al [70]	No sources received
Lin et al [71]	No sources received

Cha et al [72]	No sources received
Barr et al [73]	No sources received
Li et al [74]	No sources received
Suhara et al [75]	No sources received
Rustemović et al [76]	No sources received
Kataoka et al [77]	No sources received
Carrara et al [78]	No sources received
Ignes et al [79]	No sources received
Okasha et al [80]	No sources received
Ahmad et al [81]	No sources received
Aghaghazvini et al [82]	No sources received
Wang et al [83]	No sources received
Azizi et al [84]	No sources received
Wang et al [85]	No sources received
Kratky et al [86]	No sources received

Supplementary Table S5: PRISMA Checklist

Section and Topic	Item #	Checklist item	Location where item is reported
**TITLE**			
Title	1	Identify the report as a systematic review.	Title
**ABSTRACT**			
Abstract	2	See the PRISMA 2020 for Abstracts checklist.	Lines 16 to 34
**INTRODUCTION**			
Rationale	3	Describe the rationale for the review in the context of existing knowledge.	Lines 37 to 76
Objectives	4	Provide an explicit statement of the objective(s) or question(s) the review addresses.	Lines 76 to 80
**METHODS**			
Eligibility criteria	5	Specify the inclusion and exclusion criteria for the review and how studies were grouped for the syntheses.	Lines 85 to 96, Lines 89 to 90, Lines 95 to 104, Lines 113 to 116
Information sources	6	Specify all databases, registers, websites, organisations, reference lists and other sources searched or consulted to identify studies. Specify the date when each source was last searched or consulted.	Lines 89 to 90, supplementary table 1
Search strategy	7	Present the full search strategies for all databases, registers and websites, including any filters and limits used.	Lines 89 to 90, supplementary table 1
Selection process	8	Specify the methods used to decide whether a study met the inclusion criteria of the review, including how many reviewers screened each record and each report retrieved, whether they worked independently, and if applicable, details of automation tools used in the process.	Lines 91 to 95, lines 105 to 106
Data collection	9	Specify the methods used to collect data from reports, including how many reviewers collected data from each	Lines 104 to 109

Section and Topic	Item #	Checklist item	Location where item is reported
process		report, whether they worked independently, any processes for obtaining or confirming data from study investigators, and if applicable, details of automation tools used in the process.	
Data items	10a	List and define all outcomes for which data were sought. Specify whether all results that were compatible with each outcome domain in each study were sought (e.g. for all measures, time points, analyses), and if not, the methods used to decide which results to collect.	Lines 104 to 109, Supplementary Table 3
	10b	List and define all other variables for which data were sought (e.g. participant and intervention characteristics, funding sources). Describe any assumptions made about any missing or unclear information.	Lines 110 to 112
Study risk of bias assessment	11	Specify the methods used to assess risk of bias in the included studies, including details of the tool(s) used, how many reviewers assessed each study and whether they worked independently, and if applicable, details of automation tools used in the process.	Lines 112
Effect measures	12	Specify for each outcome the effect measure(s) (e.g. risk ratio, mean difference) used in the synthesis or presentation of results.	Lines 126 to 130
Synthesis methods	13a	Describe the processes used to decide which studies were eligible for each synthesis (e.g. tabulating the study intervention characteristics and comparing against the planned groups for each synthesis (item #5)).	Lines 116 to 118
	13b	Describe any methods required to prepare the data for presentation or synthesis, such as handling of missing summary statistics, or data conversions.	Lines 125 to 130
	13c	Describe any methods used to tabulate or visually display results of individual studies and syntheses.	Table 2
	13d	Describe any methods used to synthesize results and provide a rationale for the choice(s). If meta-analysis was performed, describe the model(s), method(s) to identify the presence and extent of statistical heterogeneity, and software package(s) used.	Lines 125 to 130
	13e	Describe any methods used to explore possible causes of heterogeneity among study results (e.g. subgroup analysis, meta-regression).	N/A
	13f	Describe any sensitivity analyses conducted to assess robustness of the synthesized results.	N/A
Reporting bias assessment	14	Describe any methods used to assess risk of bias due to missing results in a synthesis (arising from reporting biases).	N/A
Certainty assessment	15	Describe any methods used to assess certainty (or confidence) in the body of evidence for an outcome.	N/A
**RESULTS**			
Study selection	16a	Describe the results of the search and selection process, from the number of records identified in the search to the number of studies included in the review, ideally using a flow diagram.	Figure 1
	16b	Cite studies that might appear to meet the inclusion criteria, but which were excluded, and explain why they were excluded.	Figure 1
Study characteristics	17	Cite each included study and present its characteristics.	Table 2

Section and Topic	Item #	Checklist item	Location where item is reported
Risk of bias in studies	18	Present assessments of risk of bias for each included study.	Supplementary Table 2
Results of individual studies	19	For all outcomes, present, for each study: (a) summary statistics for each group (where appropriate) and (b) an effect estimate and its precision (e.g. confidence/credible interval), ideally using structured tables or plots.	Table 5 and 6
Results of syntheses	20a	For each synthesis, briefly summarise the characteristics and risk of bias among contributing studies.	N/A
	20b	Present results of all statistical syntheses conducted. If meta-analysis was done, present for each the summary estimate and its precision (e.g. confidence/credible interval) and measures of statistical heterogeneity. If comparing groups, describe the direction of the effect.	Tables 2-6
	20c	Present results of all investigations of possible causes of heterogeneity among study results.	N/A
	20d	Present results of all sensitivity analyses conducted to assess the robustness of the synthesized results.	Table 5 and 6
Reporting biases	21	Present assessments of risk of bias due to missing results (arising from reporting biases) for each synthesis assessed.	N/A
Certainty of evidence	22	Present assessments of certainty (or confidence) in the body of evidence for each outcome assessed.	N/A
**DISCUSSION**			
Discussion	23a	Provide a general interpretation of the results in the context of other evidence.	Lines 190 to 269
	23b	Discuss any limitations of the evidence included in the review.	Line 273
	23c	Discuss any limitations of the review processes used.	Lines 273 to 275
	23d	Discuss implications of the results for practice, policy, and future research.	Lines 276 to 295
**OTHER INFORMATION**			
Registration and protocol	24a	Provide registration information for the review, including register name and registration number, or state that the review was not registered.	N/A; as this was a review, it was not registered
	24b	Indicate where the review protocol can be accessed, or state that a protocol was not prepared.	N/A
	24c	Describe and explain any amendments to information provided at registration or in the protocol.	N/A
Support	25	Describe sources of financial or non-financial support for the review, and the role of the funders or sponsors in the review.	Line 304
Competing interests	26	Declare any competing interests of review authors.	Line 310
Availability of data, code and other materials	27	Report which of the following are publicly available and where they can be found: template data collection forms; data extracted from included studies; data used for all analyses; analytic code; any other materials used in the review.	Lines 307 to 308
